# Monitoring of palliative care outcomes for nursing home residents with dementia: a secondary exploratory analysis of monthly recurrent deterioration data

**DOI:** 10.3389/fpubh.2026.1874583

**Published:** 2026-09-30

**Authors:** Stefania Serban, Bogdan Neamtu, Daniela Viorica Mosoiu, Adrian Cosmin Ilie, Estera Boeriu, Maria-Mirabela Mihailescu-Marin

**Affiliations:** 1 Sibiu Municipal Hospital, Sibiu, Romania; 2 Clinical Department, Faculty of Medicine, Lucian Blaga University of Sibiu, Sibiu, Romania; 3 Research Department, Pediatric Clinical Hospital, Sibiu, Romania; 4 Faculty of Medicine, Transilvania University of Braşov, Braşov, Romania; 5 Department of Public Health and Sanitary Management, Victor Babeş University of Medicine and Pharmacy, Timişoara, Romania; 6 Center for Translational Research and Systems Medicine, Faculty of Medicine, Victor Babeş University of Medicine and Pharmacy, Timişoara, Romania; 7 Department of Pediatrics, Victor Babeş University of Medicine and Pharmacy, Timişoara, Romania; 8 Onco-Hematology Department, Louis Ţurcanu Emergency Clinical Hospital for Children, Timişoara, Romania; 9 Department of Medical and Surgical Specialties, Faculty of Medicine, Transilvania University of Braşov, Braşov, Romania; 10 HOSPICE House of Hope Foundation, Braşov, Romania

**Keywords:** dementia, IPOS-Dem, nursing homes, palliative care, predictive modeling, recurrent deterioration, secondary analysis

## Abstract

**Background:**

In long-term care facilities, regular proxy-completed multidimensional assessments may help staff detect meaningful decline in residents with dementia, including temporal changes in physical symptoms, mood, behavior, and social interaction.

**Methods:**

We conducted a secondary analysis of publicly available, de-identified longitudinal IPOS-Dem data from a Swiss stepped-wedge cluster-randomized trial to develop and evaluate a concise resident-level model predicting recurrent short-term deterioration on IPOS-Dem subscales among Swiss long-term care residents with dementia. Monthly observations were converted into prediction episodes. Deterioration was prospectively defined as a ≥0.5-point increase in DEBI (dementia emotional and behavioral impact-reflecting emotional and behavioral symptoms) or DPII (dementia physical/interaction impact indicating physical, functional, communication, and interaction-related concerns) at the next observation within 14–45 days. Recurrent deterioration was defined as deterioration in >33% of eligible episodes. Candidate logistic-regression predictors included mean DEBI/DPII, recent monthly DEBI/DPII changes, age, sex, intervention exposure, study-time variables, cluster group, and DEBI × DPII.

**Results:**

The episode-level dataset included 2,107 eligible prediction episodes from 222 residents; 535 episodes deteriorated (25.4%), and 72/222 residents (32.4%) met recurrent-deterioration criteria. In the final model, higher mean-centered DEBI increased the odds of recurrent deterioration (OR 2.655, 95% CI 1.382–5.101, *p* = 0.003), as did older age (OR 1.055/year, 95% CI 1.006–1.107, *p* = 0.027). Mean-centered dDEBI reduced risk (OR 0.089, 95% CI 0.009–0.873, *p* = 0.038). DEBI × DPII was significant (OR 0.341, 95% CI 0.118–0.991, *p* = 0.048). Performance was reasonable (AUC 0.754; Brier 0.179).

**Conclusion:**

Serial IPOS-Dem monitoring provides actionable indicators of level and trajectory for recurrent deterioration. External validation and longitudinal modeling are needed to address resident- and facility-level dependence.

## 1 Introduction

Dementia is a chronic, progressive, life-limiting syndrome marked by cognitive decline, communication difficulties, behavioral symptoms, functional dependency, and increasing care needs. According to the World Health Organization, in 2021, 57 million people worldwide were living with dementia, with nearly 10 million new cases each year (1). Projections from the Global Burden of Disease Study 2019 indicate that the number of people with dementia is expected to rise from 57.4 million in 2019 to 152.8 million by 2050 (2). In this context, dementia is increasingly recognized as a condition with substantial palliative care needs that extend beyond the final days of life (3, 4). These needs may include pain, dyspnoea, eating and swallowing difficulties, agitation, anxiety, depression, communication problems, family distress, and uncertainty around care planning (4, 5). Nonetheless, in a Cochrane review, Walsh et al. (6) concluded that evidence for palliative care interventions in advanced dementia remains limited, while Gupta et al. (5) emphasized that palliative and hospice approaches may address psychosocial and spiritual needs as well as physical ones.

Given this context, long-term care facilities represent key settings for dementia palliative care, as many residents experience frailty, multimorbidity, impaired communication, and reliance on proxy assessment. Nevertheless, unmet palliative care needs in nursing homes are often under-recognized and inconsistently reported. A 2025 scoping review by Crowley et al. (7) found substantial heterogeneity in methods, tools, and guidelines for identifying these needs and recommended routine screening to support timely access to care. Consequently, the Integrated Palliative Care Outcome Scale for People with Dementia, or IPOS-Dem, was developed to systematically assess symptoms and concerns among people with this condition in long-term care. Ellis-Smith et al. (8) found IPOS-Dem to be a comprehensive and acceptable caregiver-reported measure, while a later process evaluation identified mechanisms including improved observation, collaborative assessment, systematic record-keeping, monitoring, care planning, and communication (9).

The Swiss-German easy-language version of IPOS-Dem was developed by Spichiger et al. (10) for proxy assessment by frontline staff and family members in Swiss nursing homes. In that adaptation study, the instrument was translated and culturally adapted for the Swiss nursing-home context, and cognitive interviews supported its face and content validity. The trial protocol and main findings have been reported previously (11, 12). Subsequent psychometric work that followed provided a balanced rationale for the present analysis. The Swiss inter-rater reliability study reported variable item-level agreement and concluded that the Swiss easy-read IPOS-Dem is promising but requires further improvement before high-stakes research or decision-making (13). Conversely, the structural validity study supported the use of subscale-level information, identifying Dementia Physical and Interaction Impact and Dementia Emotional and Behavioral Impact as meaningful domains with acceptable internal consistency (14).

Building on this measurement and trial context, the present study used the publicly available trial dataset (15) to ask a different, exploratory question: whether repeated IPOS-Dem subscale measurements can identify residents at risk of recurrent short-term deterioration.

Within this framework, our research approach is a secondary exploratory analysis using data from the Swiss IPOS-Dem stepped-wedge cluster-randomized trial. Our design asks whether repeated IPOS-Dem measurements can predict which residents are at risk of recurrent short-term deterioration. Specifically, we aimed to: (1) prospectively define short-term deterioration from one monthly assessment to the next; (2) summarize deterioration at episode and resident levels; (3) estimate associations between current symptom burden, recent trajectory, and recurrent deterioration; and (4) evaluate discrimination, classification performance, and calibration of a concise logistic regression model. To the best of our knowledge, recurrence-based longitudinal deterioration endpoints remain scarcely studied in dementia palliative care (16, 17).

## 2 Materials and methods

### 2.1 Study design and data source

This brief research report analyzed publicly available, de-identified longitudinal records derived from the stepped-wedge cluster-randomized study conducted in Swiss nursing homes (15). Raw IPOS-Dem data comprised 3,176 assessment-level observations across 12 variables, each corresponding to a single resident assessment at a specific time point. Variables available for analysis included: stepped-wedge cluster group identifier (cluster_group_id; groups 1, 2, or 3), nursing home or facility cluster identifier (cluster_id), anonymized resident identifier (person_id), death indicator (death; 0/1), sex (sex; 0/1, later recoded as Female/Male), cluster cross-over day from control to intervention (cross_over_d), age in years (age), time in study in days (TInStudy), time in intervention in days (TInInt), intervention status at the time of assessment (intervention; later recoded as Control/Intervention), Dementia Physical and Interaction Impact subscale score (DPII), and Dementia Emotional and Behavioral Impact subscale score (DEBI). DEBI and DPII are IPOS-Dem subscale scores ranging from 0 to 4, with lower scores indicating lower impact and higher scores indicating greater impact. Based on the Swiss-German easy-read frontline-staff structural validity study, DPII reflected physical, functional, communication, and interaction-related concerns, whereas DEBI reflected emotional and behavioral symptoms, including agitation, wandering, anxiety, depressed mood, hallucinations or delusions, sleep problems, inner peace, and irritability or aggressiveness (14). Acceptable internal consistency was reported for both subscales, with Cronbach's α = 0.83 for DPII and α = 0.81 for DEBI (14). To the best of our knowledge, no validated clinical cut-offs or minimally important difference values are currently established for these subscales. Therefore, DPII and DEBI were analyzed as continuous predictors, and our deterioration thresholds were treated as exploratory operational definitions.

### 2.2 Analytic workflow

The longitudinal IPOS-Dem assessments were reorganized into a prediction framework linking each resident's current symptom burden and recent trajectory to deterioration at the subsequent monthly observation. For each resident, assessments were ordered chronologically. An eligible prediction episode was defined as an index visit with both a preceding assessment, used to calculate recent change, and a following assessment within 14–45 days, used to determine whether deterioration occurred at the next observation. Episode-level deterioration was coded when either DEBI or DPII increased by at least 0.5 points at the subsequent assessment. Because residents could contribute multiple eligible episodes, the final predictive model was constructed at the resident level. For each resident, we calculated the proportion of eligible episodes followed by deterioration, termed the deterioration rate (det_rate), and defined recurrent deterioration as det_rate >0.33. This aggregation reduced within-resident dependence and focused the outcome on repeated instability across follow-up rather than isolated monthly worsening.

### 2.3 Episode-level deterioration outcome

For each resident, observations were ordered chronologically by study time (TInStudy). We calculated previous values and recent changes (Equations 1, 2):


$$\begin{array}{l} dDEBI_{it}=DEBI_{it}-DEBI_{i,t-1} \end{array}$$ (1)

$$\begin{array}{l} dDPII_{it}=DPII_{it}-DPII_{i,t-1} \end{array}$$ (2)

We also calculated the next observed DEBI and DPII values and the time interval to the next observation (Equation 3):


$$\begin{array}{l} dt_{it}=TInStudy_{i,t+1}-TInStudy_{it} \end{array}$$ (3)

Prediction episodes were retained only if both previous and next values were available and if the next observation occurred within 14–45 days, approximating a monthly interval. The episode-level binary outcome was (Equation 4):


$$Y_{it}^{episode}=\{\begin{array}{cc} 1, & if\;(DEBI_{i,t+1}-DEBI_{it}\geq 0.5)\;or\;(DPII_{i,t+1}\\ \; & -DPII_{it}\geq 0.5)\\ 0, & otherwise \end{array}$$ (4)

To further evaluate the robustness of the episode-level deterioration definition, we performed sensitivity analyses using alternative point-change thresholds on the 0–4 DEBI/DPII subscale metric: ≥0.25, the primary ≥0.50 definition, ≥0.75, and ≥1.00, while keeping the resident-level recurrence definition fixed as det_rate >0.33 and retaining the same final model structure.

### 2.4 Resident-level recurrent deterioration outcome

We fitted the predictive model at the resident level, as repeated episodes within a resident were not independent. For resident *i*, the deterioration rate was (Equation 5):


$$\begin{array}{l} {det\text{\_}rate}_{i}=\frac{1}{n_{i}}\overset{n_{i}}{\underset{t=1}{\sum }}Y_{it}^{episode} \end{array}$$ (5)

The final recurrent deterioration outcome was:


$$Y_{i}=\{\begin{array}{cc} 1, & if\;det\_rate_{i}>0.33\\ 0, & if\;det\_rate_{i}\leq 0.33 \end{array}$$

The det_rate >0.33 threshold was used exploratively as a pragmatic definition of recurrence, not as a validated IPOS-Dem clinical cut-off. This approach is consistent with longitudinal outcome literature emphasizing that deterioration definitions should be clearly stated, as different definitions may yield different results (16, 17). In our dataset, residents contributed a median of 11 eligible episodes, and this threshold generally identified repeated deterioration across several monthly observations (at least four deterioration episodes) rather than isolated worsening. Its rate-based structure is methodologically consistent with the approach described in ICU delirium literature, where repeated assessments are summarized as delirium burden or the proportion of days with delirium. Nevertheless, in our dataset, the threshold identified residents with repeated deterioration across several consecutive monthly observations rather than isolated worsening (18).

To further evaluate the robustness of this pragmatic choice, we performed threshold sensitivity analyses using alternative resident-level recurrence definitions: det_rate ≥ 0.25, the primary definition det_rate > 0.33, det_rate ≥ 0.40, and det_rate ≥ 0.50, while retaining the same final model structure.

### 2.5 Predictors

Candidate predictors were selected *a priori* to represent current symptom burden (DEBI, DPII), recent trajectory (dDEBI, dDPII), resident characteristics (age,sex), and stepped-wedge design context (intervention exposure, time in study, time in intervention, interval to next observation, and cluster group), rather than through automated *p*-value-driven selection. Resident-level predictors were created by aggregating eligible episode-level data. Age and sex were treated as resident-level characteristics. DEBI, DPII, dDEBI, dDPII, time in study, time in intervention, and interval to next observation were averaged across eligible episodes for each resident. Intervention exposure was coded as “Exposed” if a resident had at least one eligible observation during the intervention period and “Control_only” otherwise. Cluster group was retained as a design-related covariate. Continuous predictors were mean-centered before fitting the interaction model.

### 2.6 Mathematical form of the logistic regression model

Let *p*_*i*_ = *P*(*Y*_*i*_ = 1) be the probability that resident *i* had recurrent deterioration. The proposed model used the logit link (Equations 6, 7):


$$\begin{array}{l} \text{logit}\left(p_{i}\right)=\log\left(\frac{p_{i}}{1-p_{i}}\right)=\eta_{i} \end{array}$$ (6)

$$\begin{array}{l} p_{i}=\frac{1}{1+\exp\left(-\eta_{i}\right)} \end{array}$$ (7)

The final linear predictor was (Equation 8):


$$\begin{array}{l} \eta_{i}=\beta_{0}+\beta_{1}DEBI_{i}^{c}+\beta_{2}DPII_{i}^{c}+\beta_{3}dDEBI_{i}^{c}+\beta_{4}dDPII_{i}^{c}\\ \;+\;\beta_{5}Age_{i}^{c}+\beta_{6}Male_{i}+\beta_{7}Exposed_{i}\\ \;+\;\beta_{8}TInStudy_{i}^{c}+\beta_{9}TInInt_{i}^{c}+\beta_{10}dt_{i}^{c}\\ \;+\;\beta_{11}ClusterGroup2_{i}+\beta_{12}ClusterGroup3_{i}\\ \;+\;\beta_{13}\left(DEBI_{i}^{c}\times DPII_{i}^{c}\right) \end{array}$$ (8)

where superscript *c* denotes mean-centered continuous predictors. Cluster group 1, female sex, and control-only intervention exposure were reference categories.

### 2.7 Statistical analysis

We summarized continuous variables as mean, standard deviation, median, range, and categorical variables as *n* (%). Normality was assessed with the Shapiro–Wilk test (*p* < 0.05 indicating non-normality). All continuous variables departed from normality at both episode and resident levels, we retained non-parametric summaries for descriptive interpretation. Resident-level model was fitted using logistic regression, with results reported as odds ratios, 95% confidence intervals, and Wald *p*-values. We assessed model discrimination using the area under the ROC curve (AUC), calibration using predicted-risk deciles, apparent calibration intercept/slope and the Hosmer–Lemeshow test and evaluated classification performance at both the conventional 0.50 threshold and the Youden–index threshold. All tests were two-sided, *p* < 0.05 was considered statistically significant, and analyses were performed in RStudio using R.

## 3 Results

### 3.1 Sample and analytic structure

Figure 1 summarizes the construction of the analytic episode-level dataset. From 3,176 raw observations, 2,107 eligible monthly prediction episodes from 222 residents remained after applying the requirement for both previous and subsequent assessments and the predefined 14–45-day next-observation window. No additional observations were excluded for missing model covariates. As shown in Table 1, residents contributed a median of 11 eligible episodes [Q1–Q3: 5–14], with a median follow-up of 11.88 months [4.76–12.67] within the eligible episode set. At resident level, mean age was 85.69 years, 72.1% were female, and the mean DEBI and DPII scores were 1.19 and 1.01, respectively.

**Figure 1:**
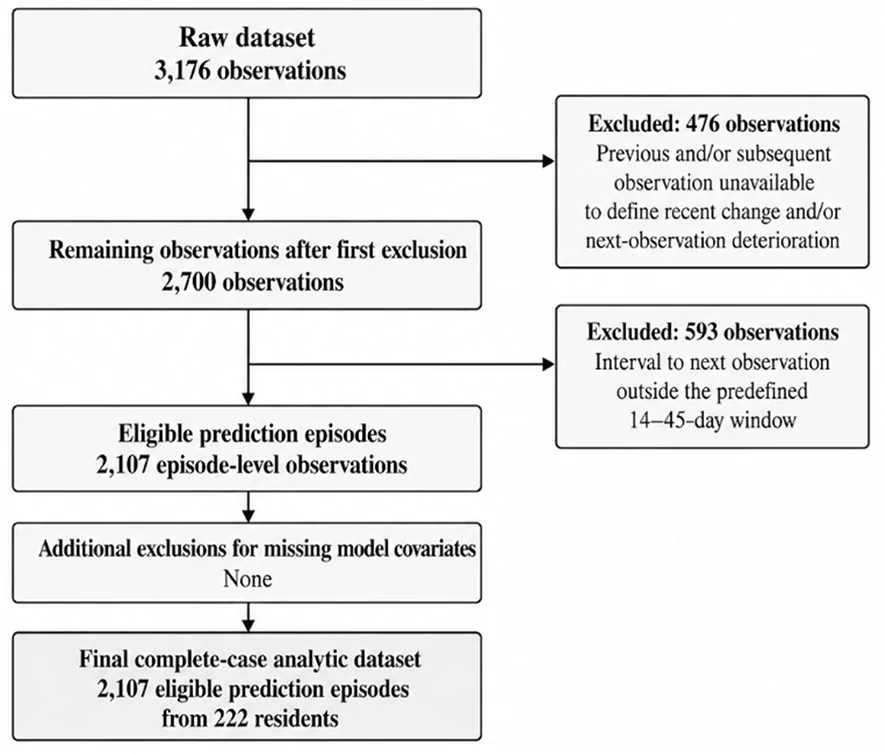
Flow diagram for derivation of the analytic episode-level dataset. Prediction episodes required information from the previous and next observation and a next-observation interval of 14–45 days. Reproduced from Spichiger and Koppitz (15).

**Table 1:** Sample and analytic dataset characteristics.

Characteristic	Value
Eligible residents, *n*	222
Eligible prediction episodes, *n*	2,107
Episode-level deterioration, *n* (%)	535 (25.4%)
Resident-level recurrent deterioration, *n* (%)	72 (32.4%)
Age, mean (SD), years	85.69 (7.86)
Age, median (range), years	86 (38–102)
Female residents, *n* (%)	160 (72.1%)
Male residents, *n* (%)	62 (27.9%)
Episodes per resident, median [Q1; Q3]	11 (5, 14)
Follow-up in eligible episode set, months, median [Q1; Q3]	11.88 [4.76; 12.67]
Mean DEBI, mean (SD)	1.19 (0.57)
Mean DPII, mean (SD)	1.01 (0.50)
Mean interval to next observation, days, mean (SD)	28.40 (2.67)

### 3.2 Episode-level and resident-level patterns

At the resident level, recurrent deterioration, defined as deterioration in more than one-third of eligible episodes (det_rate ≥ 0.33), was observed in 72 of 222 residents (32.4%). Remaining residents (150) were classified as non-recurrent deteriorators (67.6%). As shown in Supplementary Table 1, recurrent deteriorators had a higher emotional-behavioral burden, with DEBI mean 1.33 (SD 0.52), median 1.31 [0.96; 1.56], vs. 1.12 (SD 0.59), median 1.08 [0.67; 1.55], in non-recurrent residents (Mann–Whitney *p* = 0.006). DPII was also higher in the recurrent deterioration group, 1.08 (SD 0.41), median 1.04 [0.82; 1.26], vs. 0.98 (SD 0.53), median 0.90 [0.58; 1.28], but this difference was borderline (*p* = 0.069). Recent DEBI change was more negative among recurrent deteriorators, −0.03 (SD 0.20) vs. 0.02 (SD 0.16), suggesting greater emotional-behavioral instability, although the univariable association was also borderline (*p* = 0.061). The dDPII variable did not differ meaningfully between groups (*p* = 0.325). Recurrent deteriorators were slightly older, 87.10 vs. 85.02 years (*p* = 0.062), and had shorter average time in study and time in intervention (*p* = 0.027 and *p* = 0.016, respectively), while the interval to the next observation was very close, 28.49 vs. 28.35 days (*p* = 0.474). Overall, these exploratory resident-level comparisons indicate that recurrent deterioration was most consistently associated with higher DEBI burden, with weaker evidence for higher DPII and trajectory differences. These tests were unadjusted for clustering and did not account for covariates simultaneously, hence, the final multivariable logistic regression model was used as a primary adjusted predictive analysis.

Threshold sensitivity analyses showed that the recurrence definition affected event counts and apparent model performance (Supplementary Table 2). Using det_rate ≥ 0.25 produced the most inclusive definition, classifying 113 of 222 residents as recurrent deteriorators. The primary det_rate > 0.33 definition classified 72 residents, while the stricter det_rate ≥ 0.40 and det_rate ≥ 0.50 definitions classified 43 and 24 residents, respectively. Apparent discrimination remained acceptable across threshold definitions, but the stricter thresholds produced smaller event groups and were interpreted cautiously. Overall, the primary det_rate > 0.33 definition represented a pragmatic compromise between clinical recurrence and statistical stability.

At the episode level, the ≥ 0.50 threshold was retained as an exploratory intermediate definition for DEBI or DPII because it balanced interpretability and event stability (Supplementary Table 3). It represented a visible half-point change on the 0–4 DEBI/DPII subscale metric and produced an intermediate number of deterioration episodes: 535 of 2,107 episodes (25.4%). By contrast, the lower ≥ 0.25 threshold classified 892 of 2,107 episodes (42.3%) as deteriorations, suggesting potential over-inclusiveness and sensitivity to minor fluctuations. Stricter thresholds identified substantially fewer deterioration episodes: 281 of 2,107 episodes (13.3%) for ≥0.75 and 144 of 2,107 episodes (6.8%) for ≥1.00. Although these stricter thresholds showed higher apparent AUCs, the estimates were based on fewer events and were therefore interpreted cautiously as potentially less stable or overoptimistic.

### 3.3 Logistic regression model

Our model retained the DEBI × DPII interaction (Table 2). Higher mean-centered DEBI was associated with higher odds of recurrent deterioration (OR 2.655, *p* = 0.003). Older age was also associated with higher odds (OR 1.055 per year, *p* = 0.027). Mean-centered dDEBI was inversely associated with recurrent deterioration (OR 0.089, *p* = 0.038), indicating that recent emotional-behavioral trajectory contributed information beyond the average DEBI level. DPII alone was not statistically significant after adjustment, but the DEBI × DPII interaction was significant (OR 0.341, *p* = 0.048). Male sex and intervention exposure were not significant predictors in this predictive model. Cluster group contributed to the final model as well. Compared with cluster group 1, cluster group 2 was associated with lower adjusted odds of recurrent deterioration (OR 0.269, 95% CI 0.103–0.703, *p* = 0.007), whereas cluster group 3 did not differ significantly from cluster group 1 (OR 1.089, 95% CI 0.350–3.382, *p* = 0.883). Adding cluster group improved model fit, reducing AIC from 276.24 to 266.01 and BIC from 317.07 to 313.65, with a significant likelihood–ratio test, χ^2^(2)=14.23, *p* = 0.0008. This association was interpreted as evidence of possible design- or cluster-level heterogeneity rather than as an individual-level clinical effect.

**Table 2:** The logistic regression model for resident-level recurrent deterioration.

Predictor	OR	95% CI	*p*-value
**DEBI, mean-centered**	**2.655**	**1.382–5.101**	**0.003**
DPII, mean-centered	1.300	0.588–2.877	0.517
**dDEBI, mean-centered**	**0.089**	**0.009–0.873**	**0.038**
dDPII, mean-centered	0.373	0.032–4.360	0.432
**Age, mean-centered**	**1.055**	**1.006–1.107**	**0.027**
Male sex	1.082	0.517–2.267	0.834
Ever exposed to intervention	1.878	0.511–6.902	0.343
Time in study, months, mean-centered	0.724	0.521–1.007	0.055
Time in intervention, months, mean-centered	1.088	0.626–1.891	0.764
Mean interval to next observation, days	1.121	0.967–1.299	0.130
**Cluster group 2 vs. 1**	**0.269**	**0.103–0.703**	**0.007**
Cluster group 3 vs. 1	1.089	0.350–3.382	0.883
**DEBI** **×DPII interaction**	**0.341**	**0.118–0.991**	**0.048**

Continuous predictors were mean-centered but not standardized; therefore, ORs for continuous predictors represent the change in odds per one-unit increase in the original scale after centering. For DEBI and DPII, main-effect ORs should be interpreted conditionally because the final model included a DEBI × DPII interaction. Female sex, control-only exposure, and cluster group 1 were the reference categories. Cluster group is a design variable and should not be interpreted as a clinical causal factor. Bold values indicate statistically significant associations (two-sided Wald *p* < 0.05).

The equation for the fitted linear predictor was:


$$\begin{array}{l} \eta_{i}\text{\_}hat=-0.632+0.977\;DEBI_{i}^{c}+0.262\;DPII_{i}^{c}\\ \;-\;2.420\;dDEBI_{i}^{c}-0.986\;dDPII_{i}^{c}\;+0.054\;Age_{i}^{c}\\ \;+\;0.079\;Male_{i}+0.630\;Exposed_{i}-0.323\;TInStudy_{i}^{c}\\ \;+\;0.085TInInt_{i}^{c}+0.114\;dt_{i}^{c}-1.314\;ClusterGroup2_{i}\\ \;+\;0.085ClusterGroup3_{i}\;-1.075\left(DEBI_{i}^{c}\times DPII_{i}^{c}\right) \end{array}$$ (9)

where superscript *c* denotes mean-centered continuous predictors. Female sex, control-only intervention exposure, and cluster group 1 were the reference categories. Thus, *Male*_*i*_, *Exposed*_*i*_, *ClusterGroup*2_*i*_, and *ClusterGroup*3_*i*_ are binary indicator variables.

The Hosmer–Lemeshow test showed no evidence of poor fit, χ^2^ = 4.482, df = 8, *p* = 0.811. Across deciles, observed risk generally increased with predicted risk, from 0.0% observed vs. 6.4% predicted in the lowest decile to 77.3% observed vs. 72.1% predicted in the highest decile, although intermediate deciles showed variability due to small decile sizes. The apparent calibration intercept was 0.000 and the slope was 1.000, suggesting no visible in-sample miscalibration.

Multicollinearity was not considered problematic, as all adjusted GVIF values were below 2.6.

### 3.4 Model performance

The proposed model showed promise with reasonable apparent discrimination (AUC of 0.754-Figure 2 and a Brier score of 0.179). At the conventional 0.50 threshold, the model prioritized specificity (0.913) but had lower sensitivity (0.361). At the Youden threshold of 0.290, sensitivity increased to 0.792 and negative predictive value to 0.860, while specificity was 0.613 and balanced accuracy was 0.702.

**Figure 2:**
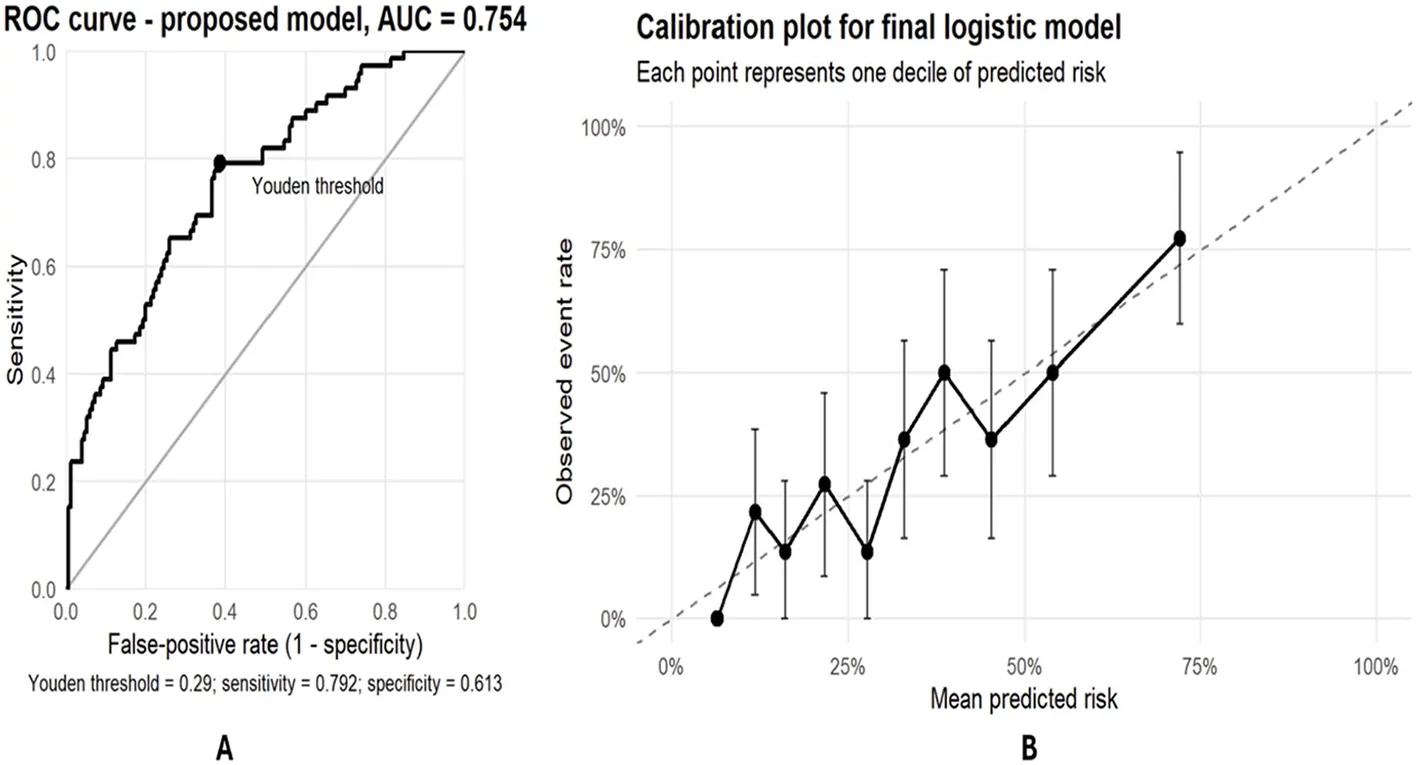
Model performance. **(A)** Receiver operating characteristic curve for the final logistic regression model, showing moderate to good apparent discrimination, AUC 0.754. **(B)** Calibration plot by predicted-risk decile, comparing observed recurrent deterioration rate with mean predicted risk, Hosmer–Lemeshow χ^2^ = 4.482, df = 8, *p* = 0.811, indicating no apparent evidence of lack of fit in this dataset.

## 4 Discussion

In long-term care, repeated monthly IPOS-Dem subscale assessments may support an exploratory level-and-trajectory framework for identifying residents with dementia at risk of recurrent short-term deterioration. Our primary conclusion shows that both the average emotional-behavioral burden and the recent trend provide valuable prognostic insights. Higher average DEBI was the clearest within-instrument signal associated with recurrent deterioration, although this association may partly reflect construct overlap because both predictors and outcome were derived from repeated IPOS-Dem subscale measurements. dDEBI and the DEBI × DPII interaction further suggested that recurrent deterioration was shaped not only by symptom level, but also by recent trajectory and the combined emotional-behavioral and physical/interaction profile. The resulting model demonstrated moderate apparent discrimination, with good sensitivity and negative predictive value at the Youden threshold, making it more appropriate for screening than for diagnostic use. Nevertheless, some model estimates presented wide confidence intervals, particularly for trajectory variables and intervention exposure, and should be interpreted cautiously. This likely reflects the modest resident-level sample size, the limited number of recurrent deterioration events, and the complexity of the final model relative to the number of events.

We further highlight the distinction between episode-level and resident-level patterns as clinically and statistically important. At the episode level, deterioration was defined as an increase of at least 0.5 points at the next monthly observation. Episodes followed by deterioration tended to have lower current DEBI and DPII and more negative prior changes. This pattern is explainable because IPOS-Dem subscales range from 0 to 4: lower current values leave more room for subsequent increases, and rebound increases may follow recent decreases. Short-term changes may also be amplified by fluctuations in proxy ratings. Our findings are consistent with the Swiss inter-rating reliability study, in which Spichiger et al. (13) reported variable item-level agreement and concluded that the Swiss easy-read IPOS-Dem is promising but requires further improvement before it can be used for high-stakes research or decision-making.

At the resident level, our results were more clinically intuitive: recurrent deterioration was associated most clearly with higher average DEBI and, less consistently, with higher DPII. This supports aggregation over repeated assessments when the objective is to identify residents with persistent instability rather than isolated monthly fluctuations. Remarkably, this resident-level interpretation aligns with the structural validity study by Spichiger et al. (14) that supports the use of subscale-level information and identifies Dementia Physical and Interaction Impact and Dementia Emotional and Behavioral Impact as meaningful IPOS-Dem domains with acceptable internal consistency.

The cluster group finding warrants cautious interpretation. It was included as a design-related covariate because it reflected the stepped-wedge sequence and may also have captured facility context and study timing. Therefore, the lower adjusted odds observed for cluster group 2 vs. cluster group 1 should not be interpreted as a protective clinical effect. More likely, it may reflect residual between-sequence or between-facility heterogeneity, including differences in resident case-mix, local care practices, implementation context, or other unmeasured cluster-level factors.

The present approach is consistent with the broader IPOS-Dem literature. IPOS-Dem was developed as a caregiver-reported measure for systematic assessment in long-term care (8), and process-evaluation evidence suggests that it can support observation, awareness, collaborative assessment, record-keeping, review/monitoring, care planning, and communication (9). The Swiss easy-language adaptation was designed to facilitate use by frontline staff and family members in nursing homes (10), while later adaptation studies emphasized the importance of cultural context, respondent interpretation, and clinical utility across settings (19, 20). A recent systematic review similarly reported initial evidence of feasibility, acceptability, and potential clinical usefulness in palliative care for dementia (23).

On the other hand, our reports complement the original intervention trial. Spichiger et al. (12) evaluated whether monthly person-centered outcome measurement followed by collaborative nurse-led person-profile discussions improved quality of life. In contrast, our analysis did not estimate the causal effect of the intervention. Rather, it examined whether repeated IPOS-Dem measurements could identify residents at risk of recurrent deterioration. Intervention exposure was retained in the model as a design-related covariate because the data came from a stepped-wedge trial and exposure status may reflect differences in study timing, post-crossover care context, and assessment conditions. Accordingly, the non-significant coefficient for intervention exposure should not be interpreted as evidence that the original intervention was ineffective. The original trial focused on quality-of-life improvement, whereas our model focused on risk stratification for recurrent symptom worsening.

Furthermore, our reports also differ from mortality prognostic tools for advanced dementia. PalS-DEM and PRO-MADE, for example, were developed to predict 6-month, 1-year, and all-cause mortality and may help with prognostication and timing of palliative care referrals (21, 22). We focused on recurrent symptom deterioration rather than survival. This distinction is clinically relevant because palliative care needs should not be addressed only when death is expected. Residents may need proactive symptom review, communication with their families, and care-plan adjustments long before they approach the end of life. It is a perspective that aligns well with Crowley et al. (7), who advocated regular screening for unmet palliative care needs in nursing homes.

Our model's performance, with an AUC of 0.754, is promising, but the internal 5-fold cross-validation suggested lower discrimination, with an AUC of approximately 0.672. The difference indicates potential optimism in the apparent performance estimate and reinforces the need for external validation before clinical use. In addition, calibration measures were derived from the development dataset and require external validation.

For context, although direct comparisons are limited by differences in populations and predicted outcomes, PRO-MADE achieved an optimism-adjusted AUC of 0.76 and a Brier score of 0.17 for 1-year all-cause mortality in hospitalized patients with advanced dementia, with the AUC decreasing to 0.70 on external validation (22). Similarly, Gebresillassie et al. (24) developed a 1-year mortality risk prediction model for older women with dementia and reported an AUC of 0.751 with good calibration after internal validation. In contrast, nursing-home mortality tools showed more modest discrimination: in a prospective bedside validation, the continuous ADEPT score predicted 6-month mortality with an AUC of 0.67, whereas Medicare hospice eligibility guidelines performed poorly, with an AUC of 0.55 (25). PalS-DEM, developed for home-dwelling patients with advanced dementia, also showed moderate discrimination for 1-year mortality, with an AUC of 0.65 (21). Together, these models provide useful benchmarks for the discrimination achieved in dementia prognostic research. However, they predict mortality, whereas the present model focuses on recurrent symptom deterioration. Comparisons must therefore account for differences in outcome, setting, and intended use. Recurrent deterioration may thus be viewed as a complementary outcome for symptom monitoring and care planning in palliative dementia care, rather than a direct substitute for mortality prognostication.

Several limitations should be acknowledged. First, both the episode-level and resident-level outcome definitions were exploratory. A validated minimally important difference has not yet been established for DEBI or DPII. Consequently, the 0.5-point increase used to define episode-level deterioration should be interpreted as an operational threshold based on its balanced interpretability and event stability rather than a validated clinical cut-off. Similarly, the resident-level recurrence definition, det_rate > 0.33, was selected pragmatically to balance clinical interpretability with event stability, but it is not an established IPOS-Dem threshold. Sensitivity analyses using det_rate ≥ 0.25, det_rate > 0.33, det_rate ≥ 0.40, and det_rate ≥ 0.50 showed that event counts varied substantially across definitions, particularly with stricter thresholds. Although resident-level aggregation reduced within-resident dependence, it did not fully model the original hierarchical data structure. Future studies should therefore evaluate both episode-level and recurrence-based deterioration definitions against external clinical anchors, such as staff concern, care-plan changes, acute clinical deterioration, hospitalization, mortality, or family-reported worsening. To this end, future work should employ longitudinal modeling strategies, such as mixed-effects logistic regression, generalized estimating equations, recurrent-event models, or joint trajectory models, to account more for repeated measurements and nursing-home-level clustering. Second, because IPOS-Dem is proxy-rated, assessments may reflect both resident status and rater perception. Third, the resident-level modeling strategy reduced dependence from repeated observations but sacrificed finer episode-level temporal detail. In addition, the final model did not include random effects for nursing homes or residents. Finally, all performance estimates were apparent and require external validation on larger datasets to confirm robust predictive utility.

Despite these limitations, the analysis provides a coherent and clinically useful framework. Repeated IPOS-Dem assessment should not be interpreted only as a static symptom score. Instead, a level-and-trajectory approach may better capture the fluctuating and multidimensional course of dementia-related palliative care needs.

## 5 Conclusion

This exploratory approach, using monthly IPOS-Dem data from Swiss long-term care facilities, supports repeated IPOS-Dem monitoring as a potential screening aid for identifying residents who may benefit from proactive case review, symptom reassessment, and person-centered palliative care planning. Future work should validate the model externally, evaluate alternative deterioration thresholds, and test mixed-effects or longitudinal prediction models that more fully account for repeated measurements and facility-level clustering.

## Data Availability

Publicly available datasets were analyzed in this study. This data can be found here: https://doi.org/10.5281/zenodo.4008427. [Spichiger and Koppitz (15); version v1].
