# Supplementary material for: Monitoring of palliative care outcomes for nursing home residents with dementia: a secondary exploratory analysis of monthly recurrent deterioration data
Source: Front Public Health. 2026 Sep 30;14:1874583. doi: 10.3389/fpubh.2026.1874583 (PMC13664699; doi:10.3389/fpubh.2026.1874583)
Supplement: Supplementary file 1 [file Table_1.docx]

Supplementary Material

**Table 1_Supplement. Resident-level numerical variables stratified by recurrent deterioration status**

| Variable | No mean(SD) | Yes mean(SD) | No median [Q1;Q3] | Yes median [Q1;Q3] | Effect (r_rb) | Magnitude | p (Mann-Whitney)*^1^* |
| --- | --- | --- | --- | --- | --- | --- | --- |
| age | 85.02 (7.92) | 87.10 (7.60) | 86.00 [81.00; 90.00] | 87.50 [83.00; 92.00] | -0.332 | Moderate | 0.062 |
| **TInStudy_months** | **5.27 (2.29)** | **4.52 (2.41)** | **6.43 [3.30; 6.76]** | **5.46 [2.50; 6.64]** | **-0.671** | **Large** | **0.027** |
| **TInInt_months** | **1.19 (1.05)** | **0.86 (1.05)** | **0.90 [0.00; 2.09]** | **0.49 [0.00; 1.36]** | **-0.684** | **Large** | **0.016** |
| **DEBI** | **1.12 (0.59)** | **1.33 (0.52)** | **1.08 [0.67; 1.55]** | **1.31 [0.96; 1.56]** | **-0.259** | **Low** | **0.006** |
| DPII | 0.98 (0.53) | 1.08 (0.41) | 0.90 [0.58; 1.28] | 1.04 [0.82; 1.26] | -0.336 | Moderate | 0.069 |
| dDEBI | 0.02 (0.16) | -0.03 (0.20) | 0.00 [-0.03; 0.05] | -0.03 [-0.11; 0.08] | -0.642 | Large | 0.061 |
| dDPII | 0.04 (0.20) | -0.00 (0.15) | 0.01 [-0.04; 0.05] | 0.01 [-0.06; 0.07] | -0.568 | Large | 0.325 |
| dt_days | 28.35 (2.79) | 28.49 (2.43) | 29.00 [27.78; 29.71] | 29.30 [27.55; 30.18] | -0.427 | Moderate | 0.474 |
| *^1^*Each person appears only once: longitudinal variables are averaged across episodes. Mann-Whitney p-values are exploratory and do not explicitly adjust for clustering. *N=222 residents; No=150, Yes=72. .* Mean(SD), Median[IQR], Mann-Whitney p and size effect. *Recurrent deterioration was defined as deterioration in more than one-third of eligible episodes (det_rate >0.33).* | | | | | | | |

**Table 2_Supplement Sensitivity analysis for recurrent deterioration threshold**

| Threshold definition | N | No | Yes | Event rate | AIC | AUC | Brier | Youden threshold | Accuracy | Sensitivity | Specificity | Balanced accuracy | PPV | NPV |
| --- | --- | --- | --- | --- | --- | --- | --- | --- | --- | --- | --- | --- | --- | --- |
| det_rate >= 0.25 | 222 | 109 | 113 | 0.509 | 300.390 | 0.716 | 0.212 | 0.484 | 0.680 | 0.770 | 0.587 | 0.679 | 0.659 | 0.711 |
| det_rate > 0.33 | 222 | 150 | 72 | 0.324 | 266.011 | 0.754 | 0.179 | 0.290 | 0.671 | 0.792 | 0.613 | 0.702 | 0.496 | 0.860 |
| det_rate >= 0.40 | 222 | 179 | 43 | 0.194 | 207.228 | 0.780 | 0.127 | 0.211 | 0.712 | 0.698 | 0.715 | 0.706 | 0.370 | 0.908 |
| det_rate >= 0.50 | 222 | 198 | 24 | 0.108 | 131.891 | 0.887 | 0.071 | 0.125 | 0.820 | 0.833 | 0.818 | 0.826 | 0.357 | 0.976 |
| Sensitivity analyses used the same resident-level final model structure, including the DEBI × DPII interaction. The 0.33 row corresponds to the primary analysis. Performance metrics are apparent and should be interpreted cautiously, especially for sparse event definitions. AIC values are shown descriptively but were not used to compare thresholds, because each threshold defines a different binary outcome. | | | | | | | | | | | | | | |

**Table 3_Supplement Sensitivity analysis for episode-level threshold**

| Episode threshold | N episodes | Episode No | Episode Yes | Episode event rate | N residents | Resident No | Resident Yes | Resident event rate | AIC | AUC | Brier | Youden threshold | Accuracy | Sensitivity | Specificity | Balanced accuracy | PPV | NPV |
| --- | --- | --- | --- | --- | --- | --- | --- | --- | --- | --- | --- | --- | --- | --- | --- | --- | --- | --- |
| Increase >= 0.25 | 2107 | 1215 | 892 | 0.423 | 222 | 53 | 169 | 0.761 | 211.873 | 0.809 | 0.126 | 0.690 | 0.806 | 0.840 | 0.698 | 0.769 | 0.899 | 0.578 |
| Increase >= 0.50 (primary) | 2107 | 1572 | 535 | 0.254 | 222 | 150 | 72 | 0.324 | 266.011 | 0.754 | 0.179 | 0.290 | 0.671 | 0.792 | 0.613 | 0.702 | 0.496 | 0.860 |
| Increase >= 0.75 | 2107 | 1826 | 281 | 0.133 | 222 | 197 | 25 | 0.113 | 129.164 | 0.895 | 0.069 | 0.167 | 0.856 | 0.840 | 0.858 | 0.849 | 0.429 | 0.977 |
| Increase >= 1.00 | 2107 | 1963 | 144 | 0.068 | 222 | 211 | 11 | 0.050 | 74.477 | 0.955 | 0.031 | 0.084 | 0.910 | 0.909 | 0.910 | 0.910 | 0.345 | 0.995 |
| The primary episode-level definition used a >=0.50-point increase in DEBI or DPII. Resident-level recurrent deterioration was kept constant as det_rate >0.33. AIC values are descriptive only because each row uses a different outcome definition. | | | | | | | | | | | | | | | | | | |
